# San Huang Shel Shin Tang beta-cyclodextrin complex augmented the hepatoprotective effects against carbon tetrachloride-induced acute hepatotoxicity in rats

**DOI:** 10.1186/s12906-016-1127-8

**Published:** 2016-05-27

**Authors:** Yu-Lan Yeh, Wei-Jen Ting, Wei-Wen Kuo, Hsi-Hsien Hsu, Yueh-Min Lin, Chia-Yao Shen, Chung-Ho Chang, Viswanadha Vijaya Padma, Yuhsin Tsai, Chih-Yang Huang

**Affiliations:** Department of Pathology, Changhua Christian Hospital, Changhua, 50006 Taiwan; Department of Medical Technology, Jen-Teh Junior College of Medicine, Nursing and Management, Miaoli, 35664 Taiwan; Graduate Institute of Basic Medical Science, China Medical University and Hospital, No. 91, Hsueh-Shih Road, Taichung, 40402 Taiwan; Department of Biological Science and Technology, China Medical University, Taichung, 40402 Taiwan; Division of Colorectal Surgery, Mackay Memorial Hospital, Taipei, 10449 Taiwan; Department of Nursing, MeiHo University, Pingtung, 91201 Taiwan; Institute of Cellular and System Medicine, National Health Research Institutes, Zhunan Town, 35053 Taiwan; Department of Biotechnology, Bharathiar University, Coimbatore, 641 046 India; School of Chinese Medicine, China Medical University, No. 91 Hsueh-Shih Road, Taichung, 40402 Taiwan; Department of Health and Nutrition Biotechnology, Asia University, Taichung, 41354 Taiwan

**Keywords:** San Huang Shel Shin Tang (SHSST), Beta-cyclodextrin, Silymarin, CCl_4_, Liver protection

## Abstract

**Background:**

San Huang Shel Shin Tang (SHSST) is a traditional herbal decoction used as a hepato-protective agent and is composed of *Rheum officinale* Baill, *Scutellaria baicalnsis* Geprgi and *Coptis chinensis* Franch (2:1:1 w/w). Beta-cyclodextrin (β-CD) modification may potentially increase the solubility and spectral properties of SHSST.

**Methods:**

In this research, the hepato-protective effects of unmodified SHSST, β-CD modified SHSST complex (SHSSTc) and silymarin were evaluated in carbon tetrachloride (CCl_4_) induced acute hepatotoxicity in rats.

**Results:**

SHHSTc (40 mg/kg/day) and silymarin (100 mg/kg/day) both decreased the CCl_4_-induced cirrhosis pathway-related transforming growth factor beta (TGF-β) and apoptosis pathway-related caspase-8 protein expressions, but SHSST (40 mg/kg/day) did not reduce TGF-β and caspase-8 significantly . Moreover, SHHSTc (40 mg/kg/day) enhanced the activation of insulin-like growth factor 1 receptor (IGF1R) mediated survival pathway than the silymarin (100 mg/kg/day) to protect the liver from damage induced by CCl_4_.

**Conclusions:**

β-CD modification promotes hepato-protective effects of SHSST and reduces the required-dosage of the SHSST.

## Background

Hepatic fibrosis is one of the most critical pathological features of chronic liver disease. Liver fibrosis is associated with the inflammatory and reparative phase of hepatic fibrosis by activated hepatic stellate cells (HSC) and can be characterized by an excessive deposition of extracellular matrix (ECM) components in the liver parenchyma [1]. Liver fibrosis is known to be a serious chronic disease and it is difficult to treat with an aggressive treatment due to possible cirrhosis outcomes. This makes it necessary to develop a new complex or cocktail of more efficient drugs with sufficiently low dosages to prevent side effects.

SHSST is a cocktail of traditional herbal decoction, with reported bioactivities such as hypotension, antioxidation, anti-inflammation and cardio-protective effects [2]. Rheum was discovered to have a hepato-protective effect and can be used to treat carbon tetrachloride (CCl_4_)-induced liver injury in rats [3, 4]. *Scutellaria* and *Coptis* were also reported to elicit similar liver protection against acute hepatotoxicity [5–7]. The similar liver protection effects of *Rheum officinale, Scutellaria baicalnsis* and *Coptis chinensis* are due to the similarity in the constituent bioactive compounds such as baicalein, which is a flavonoid [8–10].

In our previous study, the tumor necrosis factor ligand superfamily member 6 (FAS) was activated in the course of CCl_4_-induced liver failure, with down stream apoptotic protein caspase-8 on Fas-associated protein with death domain (FADD) released to cytosol, causing cleavage of caspase 3 and progression of cellular apoptosis [11, 12]. CCl_4_ induced liver failure occurs through an oxidation process when CCl_4_ is transported through the vascular system to the liver and by catalysis by mixed function oxidase (MFO) such as P450, changes into methane chloride or radicals. These radicals cause protein metabolic obstruction and inflammation in the liver, producing CCl_4_ induced acute liver injury [13, 14]. Silymarin can provide an anti-oxidation function to block the CCl_4_ catalyzing process and protect the liver from CCl_4_ induced acute liver injury but not through the P450 inhibition [15]. Many flavonoid compounds play the same role with their anti-oxidative function. Baicalein in SHSST can also enhance cell survival ability through PI3K-Akt pathway activation. The active Akt can keep the Bcl-2-associated death promoter (Bad) protein in phosphorylated type and prevent cell apoptosis [16]. Here, the liver protection function of SHSST was tested and compared with silymarin.

In our previous research, beta- cyclodextrin (β-CD) increased the solubility and spectral properties of guest molecules, especially the hydrophobic drugs, without changing their intrinsic property to permeate the cell membranes [17–19]. Thus, β-CD complex synthesis with the indicated compound or herbal decoction can enhance the solubility, stability and bioavailability of drugs [20, 21]. This research evaluated the liver protection effects of the β-CD modified SHSST complex (SHSSTc), SHSST and silymarin in CCl_4_ induced acute hepato-toxicity in rats.

## Methods

### Preparation of SHSST and SHSST-β-CD complex

The SHSST was purchased from PaiAn pharmacy (Taichung, Taiwan). The SHSST-β-CD complex was prepared by coprecipitation. β-CD (70.0 g) was dissolved in distilled water (85 ml) at 70 °C in a water bath for 1 h. SHSST (10.0 g) in ethanol (15 mL) was slowly added to the β-CD solution with continuous agitation and stirred continuously for 6 h. Following that, 40 mL of ethanol was added drop wise to regulate the solubility of the hydrophobic solute in β-CD solution. The solution was then refrigerated overnight at 4 °C. The precipitated SHSSTc (SHSST-β-CD complex, SHSST: β-CD = 1: 9 in weight) was recovered by filtration and washed with ethanol to remove unencapsulated SHSST. This residue was dried in a vacuum oven at −20 °C for 48 h. The final powder was stored at 4 °C until use.

### Animal model

The animal experimental protocol was approved by the Institutional Animal Care and Use Committee (IACUC) of China Medical University (No.100-3-B, date 2010-9-1). There were 30 SD rats (300 g in body weight, aged 10 weeks) purchased from BioLASCO Taiwan Co., Ltd and were divided into 5 groups (*n* = 6 each). The groups were designated as control, CCl_4_ (Sgma-Aldrich, Taipei, Taiwan) intraperitoneal injection treatment, CCl_4_ intraperitoneal injection combined with SHSSTc (40 mg/kg/day) oral treatment, CCl_4_ intraperitoneal injection combined with SHSST oral treatment (40 mg/kg/day), CCl_4_ intraperitoneal injection combined with silymarin (Sigma-Aldrich, Taipei, Taiwan) oral treatment. CCl_4_ intraperitoneal injection (100 mg/kg/day) treatment was performed on the 7^th^ day following 6 days of pretreatment with the test materials. After 24 h CCl_4_ intraperitoneal injection, all the rats were killed by decapitation and samples were collected immediately.

### Blood biochemical analysis

Blood was collected from rat in each group during the while decapitation and measured using the blood routine examination protocol at China Medical University Hospital. The following parameters were analyzed: TC (total cholesterol), cholesterol, AST (aspartate transaminase), ALT (alanine transaminase), BUN (blood urea nitrogen), CK (creatine kinase).

### Hemotoxyline and eosin staining

Livers from rats in each group were soaked in 10 % formalin, dehydrated through graded alcohols and embedded in paraffin wax. Following that, 2 μm thick paraffin sections were cut from these paraffin-embedded tissue blocks. The tissue sections were deparaffinized by immersion in xylene and rehydrated. Sections were stained with hematoxylin and eosin (H&E), immersed in graded alcohols followed by xylene and mounted in mounting medium kit (Surgipath, Leica Biosystems, Instrument Co., Richmond, USA). Photomicrographs were obtained using Zeiss Axiophot microscopes (Taiwan Instrument Co., Taipei, Taiwan).

### Masson’s trichrome staining

Rat livers from each group were soaked in 10 % formalin, dehydrated through graded alcohols and embedded in paraffin wax. Following that, 2 μm thick paraffin sections were cut from these paraffin-embedded tissue blocks. The tissue sections were deparaffinized by immersion in xylene and rehydrated. Samples were then stained with Masson’s trichrome (MT) using HT15 Sigma Trichrome stain (Masson) kit (HT1079, Sigma-Aldrich, Taipei, Taiwan) and the procedure was according to the protocol of the kit. MT satin was used to investigate liver histological and fibrotic changes and photomicrographs were obtained using Zeiss Axiophot microscopes (Taiwan Instrument Co., Taipei, Taiwan).

### Tissue protein extraction

Liver tissue extracts from 6 rats in each group were obtained by homogenizing in a lysis buffer (0.05 M Tris–HCl, pH 7.4, 0.15 M NaCl, 0.25 % deoxycholic acid, 1 % NP-40, 1 mM EDTA) at a ratio of 100 mg tissue/1 ml buffer. The homogenates were placed on ice and then centrifuged at 13,000 rpm for 40 min. The supernatants were collected and stored at −80 °C for further experiments.

### Western blot assay

Liver tissue extract protein concentrations were determined using the Lowry protein assay. Protein samples were separated in a 12 % SDS polyacrylamide gel electrophoresis (SDS-PAGE) with a constant voltage of 75 V for 120 min. Proteins were then transferred to Hybond-C membranes (GE healthcare UK limited., Buckinghamshire, UK) using 50 volt for 3 h. PVDF membranes were incubated in 3 % bovine serum albumin (BSA) in Tris-buffered solution (TBS). Primary antibodies (Santa Cruz Biotechnology, CA, USA) were added into the membranes to recognize the respective proteins. After washing 3 times, horseradish peroxidase-labeled antibodies were then used. Last, after 3 times washed and pictures were taken with Fujifilm LAS-3000 (GE healthcare UK limited, Buckinghamshire, UK).

### Statistical analysis

The results shown are the means ± SD of three independent experiments. Statistical analysis was performed using one-way analysis of variants. The Student’s *t*-test was used for paired samples.

## Results

After 24 h CCl_4_ intraperitoneal injection treatment, the parameters of TG and TC did not show any significant change. AST and ALT were significantly expressed after CCl_4_-IP treatment within 24 (Table 1). However, 7 days pretreatments of SHSSTc and silymarin reduced the cholesterol after CCl_4_ intraperitoneal injection. ALT expression was especially reduced in SHSST, SHSSTc and silymarin treatment groups while AST expression was reduced only in the SHSSTc treatment group.

**Table 1:** Blood Biochemical analysis of the experimental rats

	Control	None	SHSSTc	SHSST	Silymarin
CCl_4_	-	+	+	+	+
AST	28.0 ± 2.5	42.9 ± 9.7^***^	25.9 ± 5.1^##^	38.6 ± 11.6	35.1 ± 3.9
ALT	27 ± 14	195 ± 20^***^	93 ± 15^###^	116 ± 19^##^	124 ± 25^##^
TC	50 ± 15	57 ± 14	50 ± 15	56 ± 17	50 ± 18
TG	45 ± 20	54 ± 19	46 ± 43	47 ± 13	46 ± 13
CK	483 ± 56	1399 ± 147^***^	998 ± 63^#^	1137 ± 85	1024 ± 72^#^
BUN	29 ± 7	35 ± 7	31 ± 6	33 ± 7	32 ± 7

All plasma samples (*n* = 6) were prepared from control group, CCl_4_-induced acute hepatotoxicity group, CCl_4_-induced acute hepatotoxicity combined SHSST treatment group, CCl_4_-induced acute hepatotoxicity combined SHSSTc treatment group, CCl_4_-induced acute hepatotoxicity combined silymarin treatment group were calculated and shown. *TC* total cholesterol (mg/dL), *TG* triacylglycerol (mg/dL), *AST* aspartate transaminase (U/L), *ALT* alanine transaminase (U/L), B*UN* blood urea nitrogen (mg/dL), *CK* creatine kinase (U/L). ( *** = *P* <0.001 compared with control group, # = *P* <0.05 compared with CCl_4_-IP group, ## = *P* <0.01 compared with CCl_4_-IP group, ### = P <0.001 compared with CCl4-IP group)

**Fig. 1:**
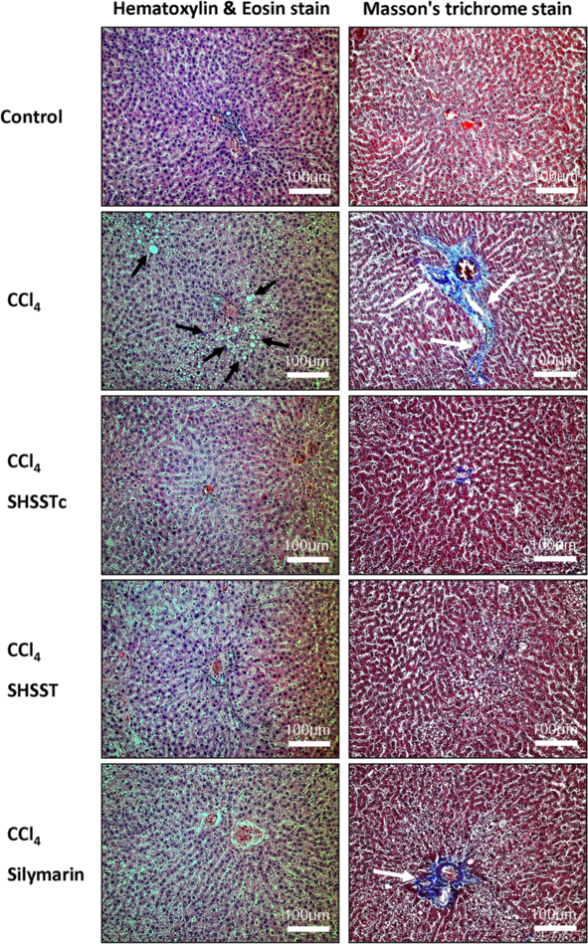
Hematoxylin and eosin stain (H&E) and Massion’s trichrome stain (MT) of liver slides. H&E stain show the CCl_4_ induce damaged group rat livers caused the lipid accumulation around the capillary and indicated by black arrows. SHSST and silymarin treatments provided a slight reduction in lipid accumulation. But SHSSTc treatment protected the liver and prevented the cell death. MT stain shows a serious liver fibrosis in CCl_4_-damaged rat liver group and are indicated by white arrows. After the silymarin, SHSST and SHSSTc treatments all can provide a great anti-fibrosis effect in CCl_4_ induced damaged rat livers. (The nuclei of the cells are stained with blue color, and others are stained by pink color by H&E staining assay. The fibrosis cells are indicated by blue color and normal cells are indicated by pink color in MT staining assay)

Further, H&E staining showed abnormal lipid accumulation within the hepatocytes around the small vascular of the liver 24 h after CCl_4_ treatment and these abnormal morphology imply the presence of fat cells with vacuolated cytoplasm which is an indication of hepatic steatosis. The SHSSTc and silymarin efficiently protected the hepatocytes and decreased the abnormality around the small vascular (Fig. 1). MT staining confirmed the fibrosis area through the collagen accumulation in CCl_4_ induced damaged liver and showed similar results in the SHSSTc and silymarin treated rat livers. SHSSTc and silymarin treatment inhibited the fibrosis and reduced the collagen accumulation in CCl_4_ induced damaged rat livers.

**Fig. 2:**
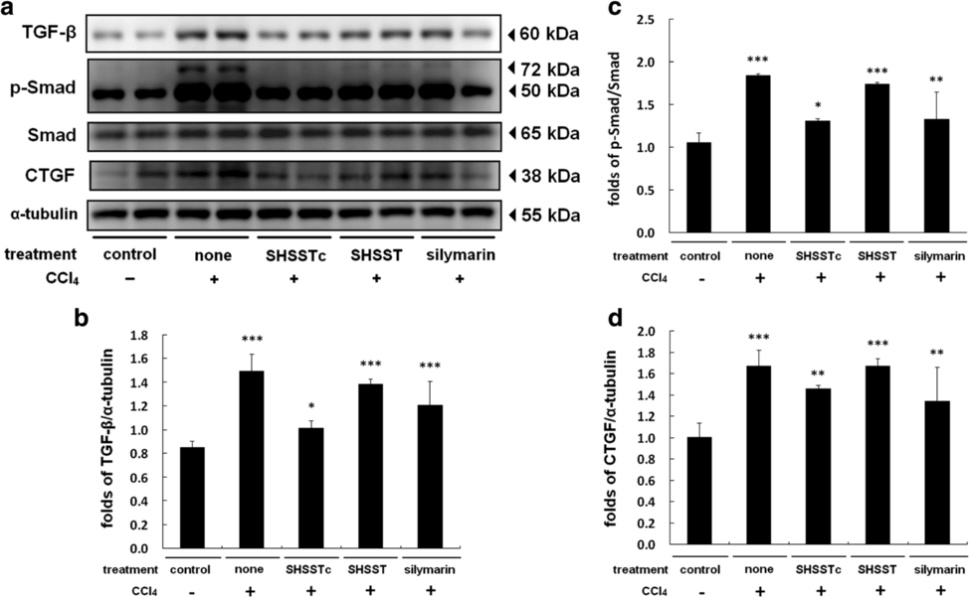
The TGF-β regulated fibrosis pathway protein expressions analysis. The protein sample of each group was analyzed by western blotting assay. **a** TGF-β/p-Smad/CTGF protein expressions increased in CCl_4_-induced acute hepato-toxicity group and reduced in SHSSTc treatment group. **b** The protein expression folds of TGF-β. **c** The protein expression folds of p-Smad. **d** The protein expression folds of CTGF. (* = *P* <0.05, ** = *P* <0.01, *** = *P* <0.001, compared with control group.)

TGF-β pathway activation is necessary for fibroblast collagen secretion in the fibrosis process. CCl_4_ intraperitoneal injection treated groups rats showed highly expressed TGF-β pathway proteins, such as p-Samd and CTGF (Fig. 2a). The TGF-β pathway protein expression showed a decrease in the SHSSTc and silymarin treated groups similar to the control group. The unmodified SHSST treated group did not show a significant reduction in TGF-β pathway protein expression. After further calculation, the SHSSTc treatment decreased TGF-β and p-Smad more efficiently than other treatments (Fig. 2b, c) however, silymarin also provided efficient reduction of CTGF in CCl_4_ induced liver fibrosis (Fig. 2d).

**Fig. 3:**
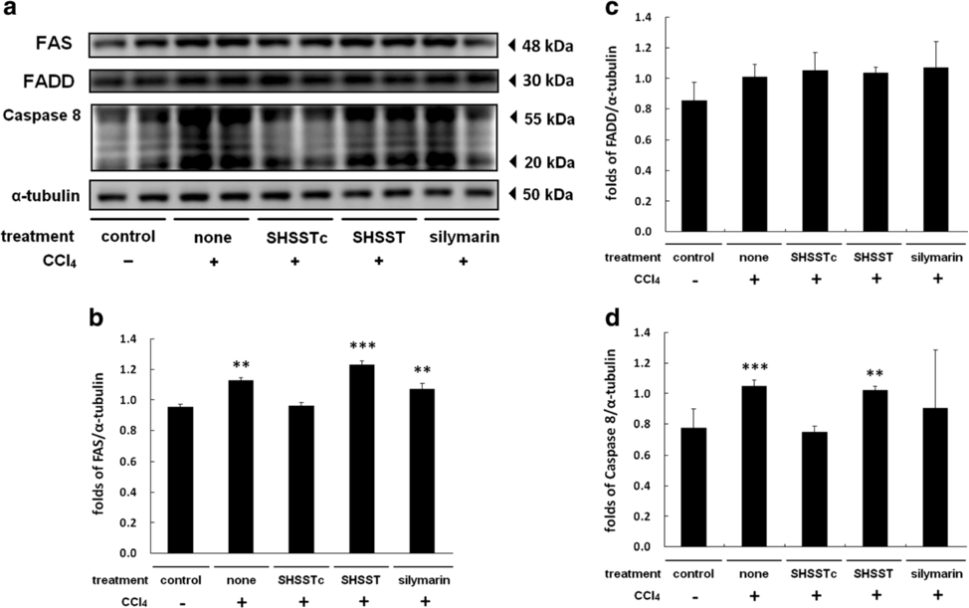
The FAS regulated apoptosis pathway protein expressions analysis. The protein sample of each group was analysis by western blotting assay. **a** FAS/FADD/Caspase 8 protein expressions increased in CCl_4_-induced acute hepatotoxicity group and decreased in SHSSTc treatment group. **b** The protein expression folds of FAS. **c** The protein expression folds of FADD. **d** The protein expression folds of Caspase 8. (* = *P* <0.05, ** = *P* <0.01, *** = *P* <0.001, compared with control group.)

FAS ligand and FAS complex are known to lead to the formation of death inducing signal complex starting with recruitment of the FAS associated death domain (FADD) adaptor protein. FADD recruits and aggregates the pro-form of caspase-8, leading to caspase-8 activation and triggering the progression of apoptosis. The liver protein analysis shows the FAS/ FADD/caspase-8 pathway activation in CCl_4_ treatment group within 24 h (Fig. 3a). Silymarin and SHSST treatment did not reduce FAS and FADD proteins by any significant level (Fig. 3b, c). However, downstream caspase-8 activation was indeed blocked by SHSSTc and silymarin treatments (Fig. 3d)

**Fig. 4:**
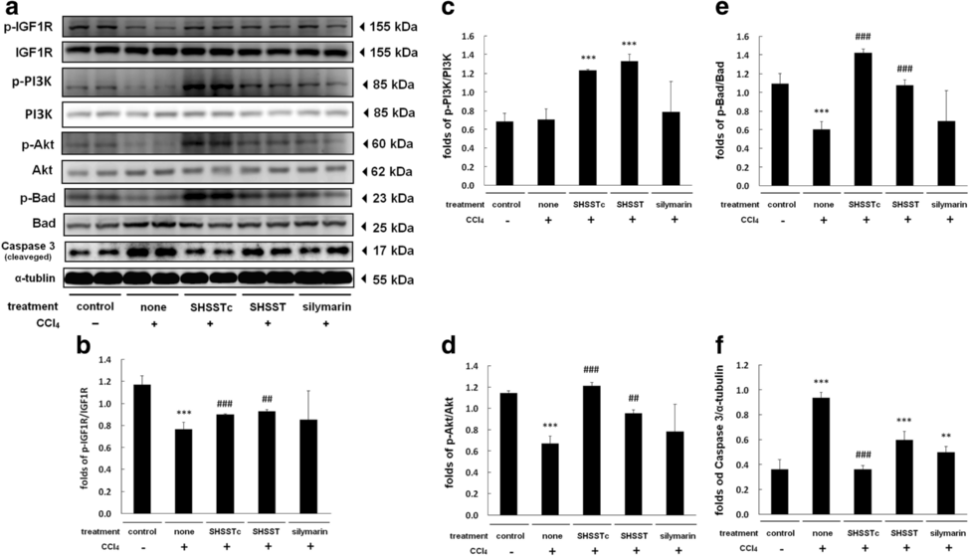
The PI3K regulated survival pathway protein expressions analysis. The protein sample of each group was analysis by western blotting assay. **a** p-IGF1R/p-PI3K/p-Akt/p-Bad protein expressions decreased in CCl_4_-induced acute hepatotoxicity group and increased in SHSSTc treatment group. Apoptotic protein Caspase 3 was increased in CCl_4_-induced acute hepatotoxicity group and decreased in SHSSTc treatment group. **b** The protein expression folds of p-IGF1R. **c** The protein expression folds of p-PI3K. **d** The protein expression folds of p-Akt. **e** The protein expression folds of p-Bad. **f** The protein expression folds of Caspase 3. (* = *P* <0.05, ** = *P* <0.01, *** = *P* <0.001, compared with control group, # = *P* <0.05, ## = *P* <0.01, ### = *P* <0.001, compared with CCl_4_ treatment group.)

The most important cell survival signaling pathway is through the activation of PI3K/Akt. In SHSSTc treatment, the p-PI3K and p-Akt were activated highly and maintained downstream Bad-phosphorylated (Fig. 4a). Finally, SHSSTc blocked caspase-3 cleavage stronger than SHSST and silymarin treatment. Both the SHSST and SHSSTc were effective than the silymarin in enhancing IGF receptor phosphorylation (Fig. 4b), and its downstream p-PI3K/p-Akt/p-Bad (Fig. 4c, d, e). The Caspase-3 expression was also partly reduced by SHSST and silymarin treatments, but was totally blocked by SHSSTc treatment (Fig. 4f).

## Discussion

CCl_4_ can be changed into CHCl_3_ through oxidation in the liver [22]. However, the products after CCl_4_ oxidation also include free radicals, such as trichloromethyl free radical (CCl_3_•) and trichloromethylperoxyl free radical (CCl_3_OO•), these free radical compounds will react with the lipoprotein in hepatocytes causing cholesterol accumulation because of the problems in lipid metabolism [23]. Silymarin can provide an anti-oxidant function and reduce the amount of free radicals in the liver, providing liver protection [24]. The flavonoids rich SHSST might also present a similar function, but the result shown the liver protection effect of SHSST was still very week (Table 1, Fig. 1). SHSSTc treatment acts better than SHSST even silymarin owing to the modulations in its bioavailability for being modified with the β-CD complex.

Silymarin is reported to provide protection against liver cirrhosis and it reduces the levels of hepatic fibrosis markers including serum TGF-β1, especially in the CCl_4_ induced liver injury model [25]. TGF-β1/p-Smad signaling expression can enhance collagen synthesis in fibroblasts [26]. The MT staining assay showed collagen accumulation in the CCl_4_ induced liver fibrosis group and TGF-β1/p-Smad signaling over expression within 24 h (Figs. 1, 2). In 1 week pretreatment with silymarin and SHSSTc, TGF-β1/p-Smad signaling and collagen accumulation were blocked (Fig. 1, 3). The experimental data suggests that SHSSTc and silymarin have similar anti- cirrhosis effects.

SHSSTc treatment efficiently protects the hepatocytes in CCl_4_ induced liver injury. The possible protection mechanism is through FAS/FADD/caspase-8 apoptosis pathway inhibition and IGF1R/PI3K/Akt survival pathway activation [27, 28] (Fig. 4). Here, silymarin could only inhibit apoptosis through caspase-8 suppression but not through IGF1R pathway activation. This may be the difference between SHSSTc and silymarin in liver protection which makes SHSSTc protect the liver better than silymarin in this CCl_4_ induced liver injury animal model.

**Fig. 5:**
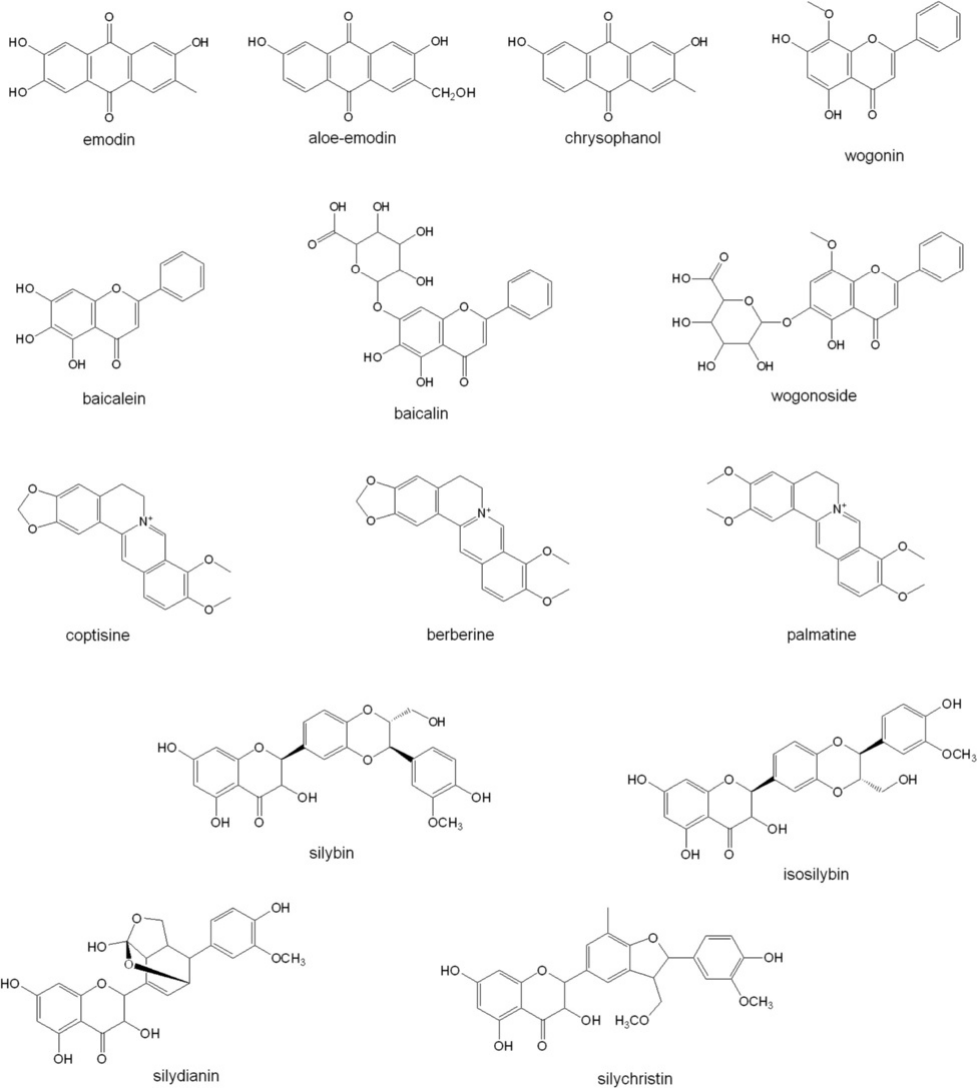
The list of bioactive compounds from SHSST and silymarin

The bioactive compounds of SHSST represent anthraquinones, such as emodin, aloe-emodin, chrysophanol, and flavones such as baicalin, baicalein, wogonoside, wogonin, and alkaloids such as berberine, palmatine, coptisine [29]. The components from silymarin are flavonols such as silybin, isosilybin, silydianin, and silychristin [30]. The chemical structures of all of these components are shown in Fig. 5. The structures of bioactive compounds from SHSST belong to the flavone and flavonol groups. Although the bioavailability of flavonol is greater than for flavones, the large functional groups in silymarin derivatives might decrease the original benefits of flavonol. The small molecular weight of the bioactive compounds from SHSST might enhance the diffusion ability. Thus, the β-CD modification can increase the water solubility of hydrophobic flavone compounds and improve the hepatoprotective effects. The SHSSTc was formed by SHSST and β-CD at the ratio of 1:9 by weight and the water solubility, stability and bioavailability were promoted better than by SHSST. Actually, both the anti-cirrhosis and liver protection effects of SHSSTc treatment were better than the other groups in the CCl_4_ induced acute liver injury animal model.

## Conclusions

SHSST modification with β-CD complex to SHSSTc enhances the liver protection effects of SHSST. SHSSTc shows similar inhibitory effects with silymarin in the FAS/FADD/caspase-8 apoptosis pathway and TGF-β fibrosis pathway. However, SHSSTc augmented the IGF1R/PI3K/Akt survival pathway more than silymarin and displayed stronger protection against CCl_4_ induced liver injury.

## Abbreviations

ALT, alanine transaminase; AST, aspartate transaminase; Bad: Bcl-2-associated death promoter; BUN, blood urea nitrogen; CCl4, carbon tetrachloride; CK, creatine kinase; ECM, extracellular matrix; FADD, Fas-associated protein with death domain; FAS, tumor necrosis factor ligand superfamily member 6; HSC, hepatic stellate cells; MFO, mixed function oxidase; SHSST, San Huang Shel Shin Tang; SHSSTc, β-CD modified SHSST complex; TC, total cholesterol; TGF-β, transforming growth factor beta; β-CD, Beta-cyclodextrin
